# Performance-based payment models and value-based health care: a semi-systematic review through theoretical lenses

**DOI:** 10.3389/frhs.2026.1816834

**Published:** 2026-08-05

**Authors:** Iara Bernz, Alessandra Rabelo, Ana Maria Malik

**Affiliations:** 1 Department of Management, São Paulo School of Business Administration, Fundação Getulio Vargas, São Paulo, SP, Brazil; 2 Department of Pharmacy, Universidade Federal de Sergipe, São Cristóvão, Brazil

**Keywords:** health policy, incentives, pay for performance, payment models, value-based health care

## Abstract

**Objectives:**

To identify and critically examine the theoretical lenses applied in the literature to conceptualize performance-based and value-based payment models, and to analyze how these frameworks explain their behavioral, organizational, and system-level effects.

**Methods:**

A semi-systematic review was conducted in July 2025 across Web of Science, PubMed/MEDLINE, Embase, LILACS, and CINAHL, using a structured search strategy and transparent selection procedures following PRISMA 2020 reporting principles. Eligible empirical and theoretical studies on value- or performance-based payment were extracted using the ADO (Antecedents, Decisions, Outcomes) framework.

**Results:**

Studies interpreted incentive effects mainly through incentive theory and principal–agent models, complemented by economics, organizational and institutional theories, implementation science, and ethics. P4P was theorized as a contractual mechanism relying on extrinsic incentives, whereas VBHC was framed as an organizational and normative paradigm requiring care redesign and longitudinal outcome–cost measurement. Theoretical extensions integrated incentive-based payment with chronic care models, disease management, activity-based costing, population health management, and the Quadruple Aim. Institutional and political economy lenses highlighted contextual contingency, while normative theories foregrounded ethical tensions, risks to professional autonomy, workforce well-being, and equity trade-offs.

**Conclusion:**

Incentive-based payment reforms are not neutral technical instruments but theory-dependent and institutionally mediated interventions that require alignment with care models, governance arrangements, and ethical safeguards.

**Systematic Review Registration:**

https://osf.io/5kv3w/, Open Science Framework (OSF), identifier 5kv3w.

## 1 Introduction

The relationship between consumers and healthcare service providers is largely mediated by financial intermediation mechanisms that operate through various models of resource allocation. These models, commonly referred to as “remuneration models” or “payment models”, define how hospitals and other healthcare providers are compensated and have long been recognized as key determinants of both healthcare expenditures and the quality of services delivered (1). Broadly, such arrangements may be structured ex ante, as in global budgeting and capitation, or ex post, as in fee-for-service and bundled payment models (1–3).

Each model has its advantages and disadvantages; however, the most widely applied in healthcare is the fee-for-service (3), which became institutionalized in the United States in the 1930s (4). In this model, each procedure is reimbursed according to an amount agreed upon between payer and provider, and payment occurs ex post, that is, after the service has been effectively delivered. Criticisms directed at this model primarily concern its tendency to encourage the overutilization of services and, consequently, to drive increases in healthcare costs (5–7).

Other payment models have gained traction over the years — including bundled payments, capitation, and global budgeting introduced both to counteract the volume-based incentives inherent in fee-for-service and to pursue broader objectives such as cost control, care coordination, and value orientation. Evidence on their effects remains mixed. While some models have shown potential to improve efficiency and support value-oriented care, notably, Maryland's all-payer global budget model and certain bundled payment arrangements have demonstrated reductions in cost growth while maintaining or improving quality in specific contexts; consistent gains in final clinical outcomes have been more difficult to demonstrate across settings. This heterogeneity suggests that the impact of payment reforms depends not only on financial incentives themselves, but also on the organizational, institutional, and behavioral mechanisms through which they operate (3, 8, 9).

As discussions shifted from service quantity and quality to the notion of value in healthcare, Porter and Teisberg (8) proposed a framework that positions patient value at the core of health system transformation. Their approach emphasizes outcomes relative to costs and rests on five fundamental components, including the organization of integrated practice units (IPUs), systematic measurement of outcomes and costs, the adoption of bundled payments for care cycles, integrated care delivery systems, expanded geographic reach, and robust information technology platforms. This value-based logic has since shaped global debates and informed the development of performance-oriented payment reforms in many countries (8).

As healthcare financing debates evolved beyond concerns with service volume and quality toward broader considerations of value, the concept of value itself became a central yet contested element of health system reform. One of the most influential formulations was proposed by Porter, who defined value as the health outcomes achieved relative to the costs required to produce them, an outcomes-to-cost ratio. This definition profoundly shaped the Value-Based Health Care (VBHC) movement and informed the design of payment reforms aimed at improving efficiency and accountability. However, this operationalization reflects a payer-centric vantage point grounded in efficiency and resource allocation logics that does not exhaust what different stakeholders regard as valuable. Patients may prioritize outcomes that are difficult to quantify, such as dignity, continuity of care, or alignment with personal goals; clinicians may emphasize professional autonomy, clinical appropriateness, and relational dimensions of care; and provider organizations may weigh sustainability, workforce well-being, and equity considerations that fall outside payer-defined metrics. Acknowledging this perspectival and contested nature of value is essential for interpreting the evidence reviewed in this study and for designing payment reforms responsive to the priorities of diverse stakeholders (10).

The literature on value-based or performance-based payment models is extensive and consists predominantly of impact evaluations (11, 12), value and performance metrics (13), expert opinions (14, 15), classifications of payment model types, and implementation frameworks (16). A substantial portion of this empirical and conceptual work is informed—explicitly or implicitly—by the framework advanced by Porter and Teisberg, which positions value as outcomes achieved relative to costs across the full cycle of care. This approach was developed in the context of the U.S. health system, characterized by fragmented service delivery, strong inter-provider competition, and a high reliance on private financing mechanisms. While influential, this dominant perspective limits the understanding of how value-based and performance-based payment models function across heterogeneous health systems, particularly those characterized by universal coverage, strong public-sector governance, or differing provider incentives.

Thus, there is a clear need to incorporate additional theoretical perspectives that explicitly address value-based and performance-based remuneration. Such perspectives are essential for more comprehensively examining the mechanisms through which these models are structured, implemented, and evaluated in diverse contexts. Expanding the conceptual repertoire is critical for identifying contextual factors that shape model performance and for informing the development of payment policies that are more responsive to the realities of the health systems in which they are intended to be applied.

In this semi-systematic review, we identify empirical and theoretical studies aimed at understanding the main theoretical lenses underpinning value-based and performance-based payment in health systems globally. We analyze the key characteristics, conceptual foundations, and rationales that support the use of these lenses to guide provider remuneration. We map the theoretical approaches described in the literature and examine major developments in the foundations of value- and performance-based payment, including those originally articulated by Porter and Teisberg. This review is guided by the following research question: Which theoretical lenses are used in the literature to conceptualize performance-based and value-based payment models in health systems, and how do these frameworks explain their behavioral, organizational, and system-level effects?

## 2 Methods

This study uses a semi-systematic review to identify and synthesize the theoretical lenses that underpin value-based and performance-based payment models in health systems. A semi-systematic approach is appropriate given the conceptual diversity of this field, where value-based payment has been defined and operationalized differently across disciplines and research traditions. Such heterogeneity limits the feasibility and relevance of a fully comprehensive systematic review (17).

Semi-systematic reviews are particularly useful for clarifying theoretical foundations and mapping the evolution of complex constructs. As described by Wong et al. (18), this approach is well-suited for topics investigated by multiple research communities and framed through varying conceptualizations. Snyder (17) further highlights that semi-systematic reviews help identify dominant themes and theoretical perspectives, synthesize the state of knowledge, map research trajectories, and outline future research agendas. These strengths align directly with the objective of the present review: to examine, organize, and interpret the theoretical lenses that inform value-based and performance-based payment models across diverse health system contexts. No formal critical appraisal of methodological quality was conducted, as the objective was interpretive and theory-oriented rather than effect estimation. This review did not aim to estimate pooled effects or determine causal impact. The review prioritizes conceptual coherence and theoretical explication over exhaustive coverage of empirical impact studies.

### 2.1 Search strategy

The search was conducted across Web of Science, PubMed/MEDLINE, Embase, LILACS, and CINAHL. The strategy was structured based on two main descriptor domains: one related to health systems and the other to payment models. The search combined controlled vocabulary (MeSH and DeCS terms) and free-text terms and included publications in English, Portuguese, and Spanish. All searches were carried out in July 2025. The full search strategy, including the descriptor table and detailed query structure, is presented in Table 1.

**Table 1:** Search strategies and database.

Databases	Search Strategy
WoS Strategy 1	TS = (“health system” OR “health systems” OR “health system agency” OR “healthcare system” OR “health care system” OR “national health system” OR “national healthcare system” OR “national health services” OR “health systems”[mesh] OR “delivery of health care"[mesh] OR “health services”[mesh] OR “health services administration”[mesh]) AND TS = (“value-based payment” OR “value-based purchasing” OR “performance-based payment” OR “alternative payment models” OR “bundled payment” OR “pay for value” OR “pay-for-value” OR “payment reform” OR “incentive payments” OR “episode-based payment” OR remuneration OR “pay-for-performance”)
WoS Strategy 2	AB = (“health system” OR “health systems” OR “health system agency” OR “healthcare system” OR “health care system” OR “national health system” OR “national healthcare system” OR “national health services” OR “health systems”[mesh] OR “delivery of health care”[mesh] OR “health services”[mesh] OR “health services administration”[mesh]) AND AB = (“value-based payment” OR “value-based purchasing” OR “performance-based payment” OR “alternative payment models” OR “bundled payment” OR “pay for value” OR “pay-for-value” OR “payment reform” OR “incentive payments” OR “episode-based payment” OR remuneration OR “pay-for-performance”)
WoS Strategy 3	TI = (“health system” OR “health systems” OR “health system agency” OR “healthcare system” OR “health care system” OR “national health system” OR “national healthcare system” OR “national health services” OR “health systems"[mesh] OR “delivery of health care"[mesh] OR “health services"[mesh] OR “health services administration"[mesh]) AND TI = (“value-based payment” OR “value-based purchasing” OR “performance-based payment” OR “alternative payment models” OR “bundled payment” OR “pay for value” OR “pay-for-value” OR “payment reform” OR “incentive payments” OR “episode-based payment” OR remuneration OR “pay-for-performance”)
LILACS	(“sistema de saúde” OR “sistemas de saúde” OR “agência do sistema de saúde” OR “sistema de atenção à saúde” OR “sistema nacional de saúde” OR “sistema de saúde pública” OR “serviços de saúde” OR “administração dos serviços de saúde” OR “prestação de cuidados de saúde”) AND (“pagamento baseado em valor” OR “compra baseada em valor” OR “pagamento por desempenho” OR “modelos alternativos de pagamento” OR “pagamento agrupado” OR “pagamento por valor” OR “reforma do pagamento” OR “pagamentos por incentivo” OR “pagamento por episódio” OR “remuneração” OR “pagamento por performance”)
LILACS	(“health system” OR “health systems” OR “health system agency” OR “healthcare system” OR “health care system” OR “national health system” OR “national healthcare system” OR “national health services” OR “health services” OR “delivery of health care” OR “health services administration”) AND (“value-based payment” OR “value-based purchasing” OR “performance-based payment” OR “alternative payment models” OR “bundled payment” OR “pay for value” OR “pay-for-value” OR “payment reform” OR “incentive payments” OR “episode-based payment” OR remuneration OR “pay-for-performance”)
PubMed Strategy 1	(“health system"[All Fields] OR “health systems"[All Fields] OR “health system agency"[All Fields] OR “healthcare system”[All Fields] OR “health care system”[All Fields] OR “national health system"[All Fields] OR “national healthcare system”[All Fields] OR “national health services”[All Fields] OR “delivery of health care”[MeSH Terms] OR “health services”[MeSH Terms] OR “health services administration”[MeSH Terms]) AND (“value-based payment"[All Fields] OR “value-based purchasing”[All Fields] OR “performance-based payment”[All Fields] OR “alternative payment models”[All Fields] OR “bundled payment”[All Fields] OR “pay for value”[All Fields] OR “pay-for-value”[All Fields] OR “payment reform”[All Fields] OR “incentive payments”[All Fields] OR “episode-based payment”[All Fields] OR “remuneration”[All Fields] OR “pay-for-performance”[All Fields]) AND [[english]/lim OR [portuguese]/lim OR [spanish]/lim]
PubMed Strategy 2	(“health systems”[tiab] OR “health system agency”[tiab] OR “healthcare system”[tiab] OR “health care system”[tiab] OR “national health system”[tiab] OR “national healthcare system”[tiab] OR “national health services”[tiab]) AND (“value-based payment”[tiab] OR “value-based purchasing”[tiab] OR “performance-based payment”[tiab] OR “alternative payment models”[tiab] OR “bundled payment”[tiab] OR “pay-for-value”[tiab] OR “payment reform”[tiab] OR “incentive payments”[tiab] OR “episode-based payment”[tiab] OR “remunerate”[tiab] OR “remunerated”[tiab] OR “remunerating”[tiab] OR “remuneration”[tiab] OR “remunerations”[tiab] OR “pay-for-performance”[tiab])
Embase Strategy 1	(“health system” OR “health systems” OR “health system agency” OR “healthcare system” OR “health care system” OR “national health system” OR “national healthcare system” OR “national health services” OR “health system”/exp OR “delivery of health care”/exp OR “health services”/exp OR “health services administration”/exp) AND (“value-based payment” OR “value-based purchasing” OR “performance-based payment” OR “alternative payment models” OR “bundled payment” OR “pay for value” OR “pay-for-value” OR “payment reform” OR “incentive payments” OR “episode-based payment” OR remuneration OR “pay-for-performance”) AND [[english]/lim OR [portuguese]/lim OR [spanish]/lim]
Embase Strategy 2	(“health system”:ti,ab OR “health systems”:ti,ab OR “health system agency”:ti,ab OR “healthcare system”:ti,ab OR “health care system”:ti,ab OR “national health system”:ti,ab OR “national healthcare system”:ti,ab OR “national health services”:ti,ab) AND (“value-based payment”:ti,ab OR “value-based purchasing”:ti,ab OR “performance-based payment”:ti,ab OR “alternative payment models”:ti,ab OR “bundled payment”:ti,ab OR “pay for value”:ti,ab OR “pay-for-value”:ti,ab OR “payment reform”:ti,ab OR “incentive payments”:ti,ab OR “episode-based payment”:ti,ab OR remuneration:ti,ab OR “pay-for-performance”:ti,ab) AND [[english]/lim OR [portuguese]/lim OR [spanish]/lim]
CINAHL Strategy 1	AB (“health system*” OR “healthcare system*” OR “health care system*” OR “national health system*” OR “health system agency” OR “national healthcare system*” OR “national health services”) AND AB (“value-based payment” OR “value based payment” OR “value-based purchasing” OR “performance-based payment” OR “alternative payment model*” OR “bundled payment*” OR “pay for value” OR “pay-for-value” OR “payment reform*” OR “incentive payment*” OR “episode-based payment*” OR remuneration OR “pay for performance”)
CINAHL Strategy 2	TI = (“health system*” OR “healthcare system*” OR “health care system*” OR “national health system*” OR “health system agency” OR “national healthcare system*” OR “national health services”) AND TI = (“value-based payment” OR “performance-based payment” OR “bundled payment*” OR “pay for value” OR “pay-for-value” OR “value-based purchasing” OR “alternative payment model*” OR “payment reform*” OR “incentive payment*” OR “episode-based payment*” OR remuneration OR “pay for performance” OR “pay-for-performance”)

Source: Developed by authors (2025).

### 2.2 Inclusion and exclusion criteria

Studies published in Portuguese, English, or Spanish and available in full text online were included, with no restrictions on the year of publication. Eligible studies comprised empirical or theoretical research with clear methodological descriptions and direct relevance to performance-based and/or value-based payment models for medical and hospital service providers. Financing approaches not specifically focused on value or performance were excluded. The selection included original research articles, systematic and narrative reviews, case studies, and qualitative or quantitative studies evaluating the implementation, performance, or impacts of such models in health systems.

Exclusion criteria include letters to the editor, commentaries, editorials, and opinions without empirical grounding, as well as conference abstracts without accessible full texts. Studies addressing global financing arrangements without a value or performance focus, and those related broadly to negotiations with the pharmaceutical industry, were excluded.

### 2.3 Screening

The Screening process was conducted in sequential stages to ensure transparency and methodological rigor, following PRISMA 2020 reporting principles. In the first stage, two reviewers independently assessed titles and abstracts for relevance using Rayyan® (Qatar Computing Research Institute), based on the predefined inclusion and exclusion criteria. Discrepancies were resolved through discussion and, when necessary, consultation with a third reviewer. After the removal of 9,713 duplicate records, 14,202 records were screened. Of these, 11,227 were excluded for reasons including wrong inclusion criteria (*n* = 3,941), publication type (*n* = 1,286), objective and remuneration focus (*n* = 5,530), or study design (*n* = 470). A total of 2,975 reports were sought for retrieval, of which 2,242 could not be retrieved. Most non-retrieved reports corresponded to conference proceedings, inaccessible full texts, or publications without institutional access despite multiple retrieval attempts.

In the second stage, 733 reports underwent full-text assessment for eligibility by two independent reviewers. The objective of this phase was to identify studies that explicitly described the theoretical lenses used to conceptualize value-based or performance-based payment models. Following full-text review, 657 reports were excluded for not meeting the eligibility criteria, resulting in 76 studies included in the final synthesis.

**Figure 1:**
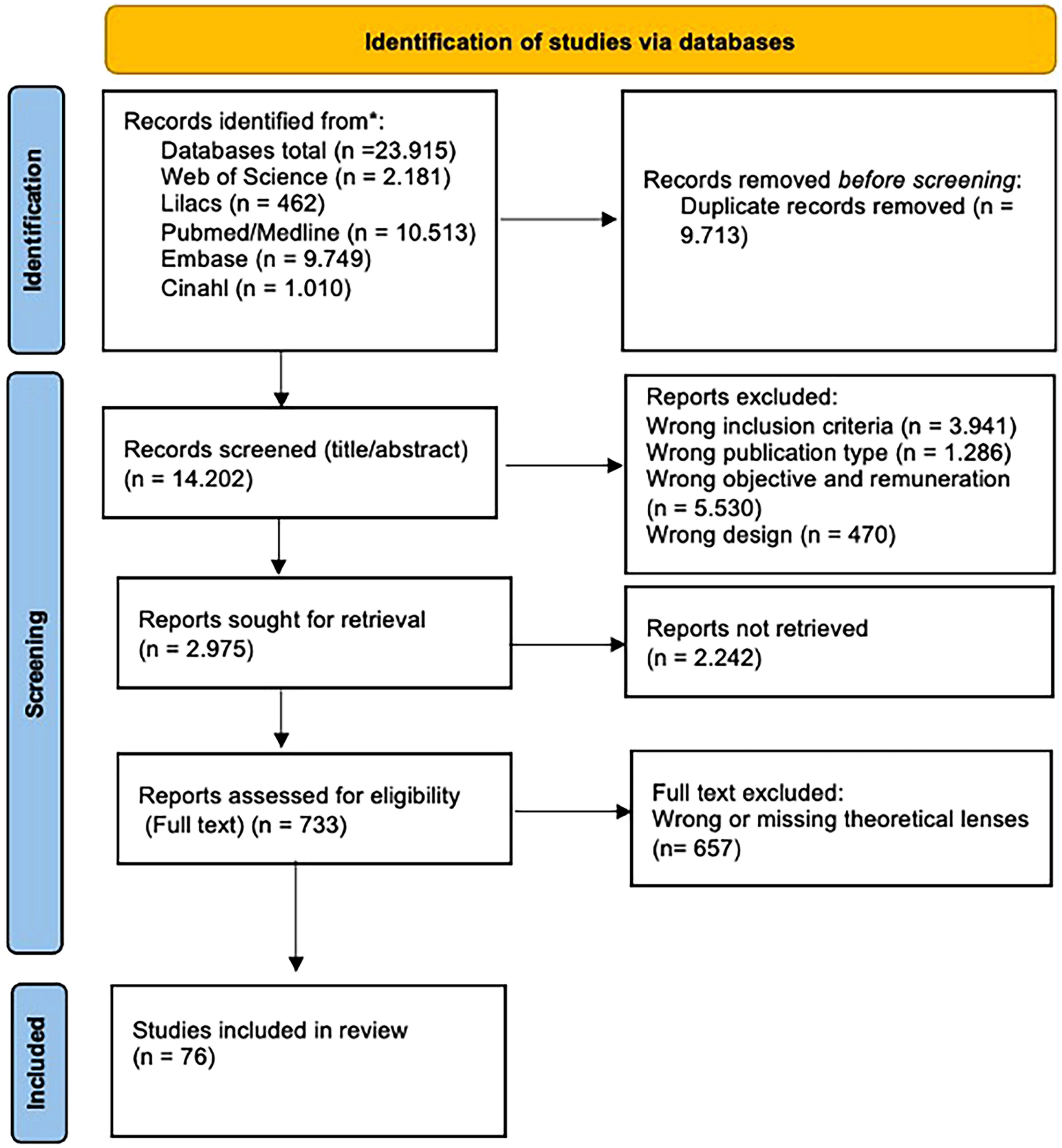
Prisma diagram. Source: Developed by authors (2025).

A detailed visualization of the study identification, screening, and inclusion steps is presented in the PRISMA flow diagram (Figure 1).

### 2.4 Data extraction

The results of this review were synthesized using thematic tables and a complementary narrative summary. Data extraction followed a structured template designed to enhance comparability across studies. Extracted information included: (i) the type of payment model examined, (ii) the theoretical lens applied, (iii) the country and level of care in which the model was implemented, and (iv) the outcomes or other system-level effects associated with the model. This approach supports cross-context comparison and enables a rigorous assessment of each study's contributions and limitations relative to the research objectives.

**Figure 2:**
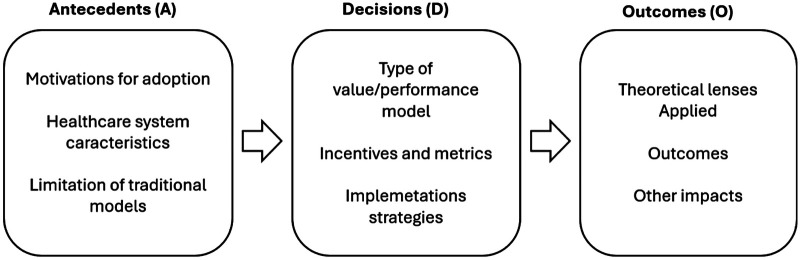
ADO framework. Source: Developed by authors (2025).

Data extraction was guided by the ADO (Antecedents, Decisions, and Outcomes) framework (Figure 2). For each included study, extracted variables were organized into thematic categories, allowing a coherent and comparative synthesis of the theoretical foundations underpinning value-based and performance-based payment models:

- Antecedents: contextual factors driving the adoption of value-based or performance-based payment models, characteristics of the health system, and limitations of traditional payment approaches.
- Decisions: the specific payment model described, associated incentive mechanisms, performance indicators, and implementation strategies.
- Outcomes: theoretical lenses applied, how these lenses informed the interpretation or justification of the model, and the outcomes reported (clinical, economic, organizational, or equity-related).

For all included studies, two reviewers independently extracted data using a standardized Microsoft Excel® data extraction form developed specifically for this review. Theoretical lenses were identified through full-text screening conducted in Rayyan®, with reviewers searching for explicit reference to a theoretical or conceptual framework, including constructs related to intrinsic and extrinsic motivation and behavioral economics. The extracted information included study characteristics, methodological design, country and healthcare setting, payment model, dominant analytical focus, key observations, and the predominant theoretical lens underpinning the study.

Following the independent extraction, reviewers compared their classifications. Disagreements regarding the identification of the predominant theoretical lens or study classification were resolved through discussion and consensus between the two reviewers. When consensus could not be reached, a third reviewer adjudicated the final classification.

In the final stage of the analysis, the identified theoretical lenses were compared according to their underlying assumptions and explanatory mechanisms. Through an iterative process of constant comparison and conceptual refinement, related theories were grouped into higher-order analytical categories, resulting in the five theoretical domains presented in the Results section.

## 3 Results

The full study-level evidence, including study design, context, payment model, theoretical framework, and reported outcomes, is provided in Supplementary Table S1.

A total of 76 studies were included, all of which explicitly named a theoretical lens or conceptual framework in the text. Of these, 56.6% (*n* = 43) were conducted in the United States. Among the remaining studies, 31.6% (*n* = 24) were produced in other high-income countries, including Western Europe, Canada, Nordic countries, and Australasia, whereas 7.9% (*n* = 6) were conducted in low- and middle-income countries, with Taiwan accounting for the largest share within this group (3.9%; *n* = 3). Brazil was represented by only two studies (2.6%; *n* = 2), indicating its marginal presence in the literature with an explicit theoretical lens. Low- and middle-income countries outside the groups described above were only residually represented (<4%; *n* = 3), comprising two studies from Sub-Saharan Africa (Tanzania) and one from China.

Corroborating the predominance of studies conducted in the United States, we observed that the health system contexts analyzed were largely those of the U.S. multiplayer system, consistent with geographic distribution noted above and primarily involved federal programs such as Medicare and Medicaid, as well as reforms driven by the Affordable Care Act, the Medicare Access and CHIP Reauthorization Act of 2015 (MACRA), and value-based payment models coordinated by the Centers for Medicare & Medicaid Services (CMS). Public or universal health systems in high-income countries outside the United States accounted for 31.6% of the studies, including the United Kingdom's National Health Service, the Canadian health system, and several European and Nordic systems. Not all studies conducted in the Global South examined universal or public systems, which explains the discrepancy between the geographic distribution (19.7%) and the classification by type of health system. Public systems in low-income countries were only residually represented, accounting for 3.9% of the studies.

Study designs were heterogeneous, with 77.0% (*n* = 58) being non-experimental studies, predominantly conceptual, normative, or policy analyses (38.5%; *n* = 29), followed by quantitative observational studies (23.1%; *n* = 18) and qualitative studies (15.4%; *n* = 11). Randomized controlled trials were rare, accounting for 2.6% (*n* = 2) of the studies.

After analyzing all eligible studies, the reported results were predominantly mixed, reflecting heterogeneity in payment designs, institutional contexts, and outcome measures. Approximately 41% (*n* = 31) of the studies reported positive results, primarily in process indicators such as adherence to clinical guidelines, monitoring, continuity of care, and care coordination, as well as in intermediate economic indicators, including episode-level cost reductions, slowed expenditure growth, and returns on investment (12, 19, 20). Observed effects were more consistent when financial incentives were combined with structured organizational models, such as disease management, the Chronic Care Model, bundled payments, or risk-adjusted capitation. Studies reporting mixed results accounted for 44% (*n* = 33) of the sample and were characterized by modest improvements in process and efficiency indicators, accompanied by inconsistent or null effects on final clinical outcomes, such as mortality and readmissions, as well as the recurrent presence of unintended effects, including gaming, narrow focus on incentivized metrics, selective spillover effects, and penalization of providers serving socially vulnerable populations (19, 21–23).

Accordingly, approximately 15.8% (*n* = 12) of the studies reported predominantly negative or critical findings, particularly those examining organizational, ethical, and equity-related impacts, highlighting the lack of reductions in disparities, crowding-out of intrinsic motivation, professional conflicts, and regressive effects of programs based exclusively on average performance without adequate social risk adjustment (24, 25). The studies indicated that process indicators and service utilization tend to respond more rapidly to incentives than hard clinical outcomes, whereas positive economic results are more likely to depend on risk protection mechanisms and governance arrangements appropriate to the payment model.

The pattern of results varied systematically across theoretical lenses. Studies grounded in incentive economics and principal-agent theory predominantly reported positive effects on process indicators and episode-level cost reductions, but also documented gaming, adverse selection, and excessive focus on contracted metrics. Studies adopting the Value-Based Health Care framework reported care redesign and transparency gains, particularly when combined with activity-based costing, though they also noted difficulties in measuring outcomes across the full care cycle. Behavioral economics and motivation theory studies identified design conditions that preserve intrinsic motivation, while also documenting crowding-out effects and reduced professional engagement. Organizational and institutional approaches highlighted that more consistent effects emerge in contexts with robust governance capacity, though significant institutional contingency limits generalizability. Studies integrating care delivery models showed more sustained effects when pay-for-performance was combined with chronic care or disease management structures. Finally, normative studies grounded in ethics, equity, and social justice consistently identified regressive effects and tensions between efficiency objectives and distributive commitments.

Table 2 organizes these findings by theoretical lens, illustrating how the pattern of results varies systematically across the conceptual frameworks examined in the following discussion.

**Table 2:** Theoretical lenses, frequency in the corpus, and reported outcome patterns (*n* = 76 studies).

Theoretical lens	*n* (%)	Predominantly positive findings	Mixed or null findings	Negative findings/unintended effects
Incentive economics and principal–agent theory	30 (39.5%)	Improvements in process indicators; protocol adherence; episode-level cost reductions	Heterogeneous effects across contexts; gains concentrated in measurable dimensions	Gaming; adverse selection (cream-skimming); excessive focus on contracted metrics
Value-Based Health Care and health value theory	18 (23.7%)	Care redesign; greater transparency; outcome improvements when combined with TDABC	Partial operationalization of value; concentration on short-term measurable outcomes	Payer-centric definition of value; difficulty measuring outcomes across the full care cycle
Behavioral economics and motivation theory	9 (11.8%)	Identification of design conditions that preserve intrinsic motivation	Heterogeneous responses depending on perceived autonomy and control	Crowding-out of intrinsic motivation; superficial compliance; reduced professional engagement
Organizational and institutional approaches	8 (10.5%)	More consistent effects in contexts with robust organizational capacity and aligned governance	High institutional contingency; limited generalizability across systems	Reconfiguration of power relations; tensions between clinical and managerial logics
Care delivery and quality models	6 (7.9%)	More sustained effects when P4P integrated with Chronic Care Model or disease management	Limited generalizability to contexts without longitudinal coordination structures	Effects restricted to process indicators without impact on final clinical outcomes
Ethics, equity, and social justice	5 (6.6%)	—	Normative tensions between efficiency and equity; risks to professional autonomy	Regressive effects; penalization of providers in vulnerable contexts; risk of care commodification

frequencies refer to theoretical lenses explicitly named in included studies. Each study was assigned to a single lens category according to the framework most extensively developed in the study's analytical body — specifically, the lens that explicitly underpins the payment model design, the incentive mechanism, or the governance framework, as described in the ‘Theoretical lens’ and “How theory underpins the model” columns of Supplementary Table S1. Of the 76 included studies, 28 (36.8%) cited at least one secondary theoretical lens alongside the primary classification; these secondary lenses are documented in Supplementary Table S1 but are not reflected in the frequencies above. Outcome patterns were synthesized narratively from reported findings within each group of studies and do not constitute a quantitative synthesis.

## 4 Discussion

This discussion is grounded in a theory-based review, following the methodological approach proposed by Kozlenkova, Samaha, and Palmatier (26). Rather than organizing the literature thematically or descriptively, this approach structures the analysis around explicit theoretical lenses, allowing for the identification of core assumptions, causal mechanisms, theoretical extensions, contextual contingencies, and normative critiques underlying the empirical and conceptual studies reviewed (Table 3).

**Table 3:** Summary of theoretical lenses, core assumptions, expected mechanisms, typical outcomes, and key references.

Theoretical lens	Core assumptions	Expected mechanisms	Typical findings across the reviewed studies	Representative references
Incentive economics & principal–agent theory	Health care is characterized by information asymmetry and potentially misaligned incentives between principals (payers/regulators) and agents (providers). Financial incentives are expected to align provider behavior with system objectives.	Performance-contingent contracts, financial rewards, penalties, and accountability mechanisms reduce opportunistic behavior and improve efficiency.	Most frequently adopted theoretical foundation. Associated with improvements in incentivized process indicators and episode-level efficiency, but also with gaming, adverse selection, multitasking, and emphasis on measurable indicators.	Holmström & Milgrom (26); Baxter et al. (21); Mathes et al. (12)
Value-Based Health Care & health value theory	Value is created by maximizing patient outcomes relative to the resources used throughout the care cycle. However, value is interpreted differently by patients, providers, and payers, although most empirical evaluations adopt a payer-centered definition.	Care redesign, integrated practice units, longitudinal outcome measurement, TDABC, bundled payments, and coordinated care delivery.	Encourages organizational transformation and transparency but remains difficult to operationalize. Most studies measure value using payer-oriented metrics, with limited incorporation of patient- or provider-defined value.	Porter & Teisberg (8, 10, 27, 28); Porter (10); French et al. (29)
Behavioral economics & motivation theory	Decision-making is influenced by bounded rationality, cognitive heuristics, framing, intrinsic motivation, and social norms rather than purely rational economic calculations.	Incentive framing (loss aversion), social comparison, peer pressure, autonomy support, and preservation of intrinsic motivation shape behavioral responses.	Explains heterogeneous responses to similar financial incentives. Well-designed incentives may enhance engagement, whereas poorly designed schemes may crowd out intrinsic motivation or generate superficial compliance.	Kahneman & Tversky (30); Deci & Ryan (31); Himmelstein & Ariely (22)
Organizational & institutional approaches	Payment reforms operate within complex organizations whose governance structures, institutional logics, and implementation capacity shape outcomes.	Leadership, organizational learning, institutional work, governance, trust, and adaptation mediate implementation.	Organizational capacity consistently moderates the effectiveness of payment reforms. Similar payment models produce different outcomes depending on governance, institutional context, and implementation maturity.	Abrahamson et al. (32); Fiss (33, 34); Reindersma et al.
Care delivery & quality improvement models	Financial incentives alone are insufficient to improve value unless embedded within coordinated models of care delivery.	Integration with Chronic Care Model, Disease Management, multidisciplinary teams, population health management, and longitudinal care coordination.	More consistent improvements occur when payment reforms are coupled with care redesign. Financial incentives alone rarely produce sustained improvements in final clinical outcomes.	Beich et al. (35); Bodenheimer & Sinsky (36)
Ethics, equity & social justice	Payment reforms are not value-neutral. Incentive systems may affect professional autonomy, distributive justice, equity, and access to care.	Incorporation of ethical safeguards, equity-sensitive performance measures, social risk adjustment, and governance mechanisms.	Identifies ethical tensions, risks of penalizing providers serving vulnerable populations, and potential commodification of care if efficiency becomes the dominant objective.	Blustein et al. (37); Chen et al. (23, 38); Buetow & Entwistle (39)

Source: Developed by authors (2026).

**Figure 3:**
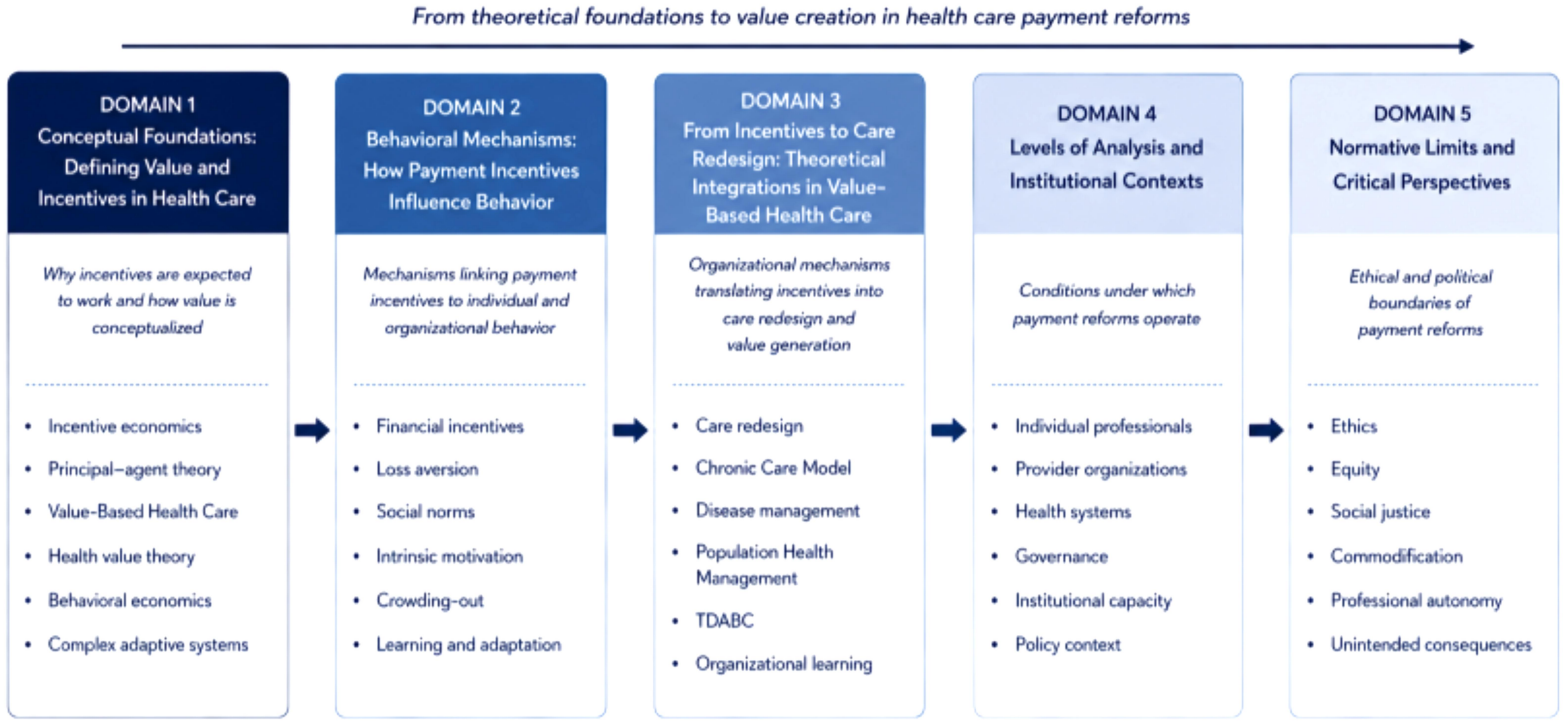
Integrative theoretical framework of payment reforms and value-based health care. Source: Developed by authors (2026).

As synthesized in Figure 3, the resulting framework organizes these theoretical contributions into five interrelated analytical domains, illustrating how different theoretical traditions explain the mechanisms through which payment reforms influence provider behavior, organizational change, and value creation in health care. Rather than representing a linear sequence, the framework highlights complementary levels of explanation that collectively support the design, implementation, and critical evaluation of performance-based payment and Value-Based Health Care models.

### 4.1 Domain 1—conceptual foundations: defining value and incentives in health care

The reviewed literature reveals a relatively coherent conceptual core, predominantly grounded in incentive economics and principal–agent theory, which underpin most analyses of performance-based payment models and Value-Based Health Care (VBHC) (21, 27, 28). These frameworks rest on the assumption that health care systems are characterized by information asymmetries and potentially misaligned objectives between principals—such as payers, regulators, and patients—and agents, including health care professionals and provider organizations. Under such conditions, contracts and incentive mechanisms are justified as tools to align behavior with system-level goals (29). The complexity of care delivery, clinical uncertainty, and the knowledge-intensive nature of medical decision-making further amplify informational asymmetries, limiting direct performance monitoring and increasing the risk of opportunistic behavior or misalignment between economic incentives and clinical objectives (21, 28).

Within this framework, the literature consistently argues that traditional retrospective payment models—most notably fee-for-service—tend to exacerbate agency problems by incentivizing service volume irrespective of value generated. In response, performance-based and value-based payment models have been advanced as contractual mechanisms designed to internalize system objectives by linking provider remuneration to indicators of quality, efficiency, or clinical outcomes (12).

Accordingly, financial incentives are conceptualized as contractual instruments intended to align interests, curb opportunistic behavior, and promote efficiency and accountability (21). Models such as pay-for-performance (P4P) and broader value-based payment schemes are thus framed as responses to the structural limitations of volume-based reimbursement systems, particularly fee-for-service arrangements that reward increased production rather than value creation. From a principal–agent perspective, tying remuneration to observable performance indicators is expected to mitigate agency problems, provided that relevant outcomes are measurable and contractible (12).

However, the application of principal–agent theory to health care exposes important limitations. Unlike industrial or financial settings, health care delivery involves multiple, interdependent, and difficult-to-measure tasks — a structural feature consistent with its characterization as a Complex Adaptive System (CAS) (30, 31). In CAS, agents operating across interdependent levels of organization produce emergent behaviors that cannot be predicted or controlled through linear incentive structures alone. Fully performance-contingent contracts are therefore inherently incomplete in this context. The multitasking problem, as articulated by Holmström and Milgrom (29), is particularly salient here: incentives tied to specific measurable dimensions of performance may induce agents to prioritize rewarded activities while neglecting equally important but less observable tasks such as care coordination, communication, and patient-centeredness, precisely the relational and emergent properties that CAS frameworks foreground as critical to system performance.

These limitations are further underscored by the incorporation of motivation theories, which distinguish between extrinsic and intrinsic motivation. Health care professionals are motivated not only by financial rewards but also by professional norms, ethical commitments, and identification with patient welfare. This distinction between extrinsic and intrinsic motivation is central to understanding how incentive design shapes professional behavior, a dynamic explored in greater depth in Domain 2.

Within the Value-Based Health Care paradigm, principal–agent theory remains central but is reinterpreted. The analytical focus shifts from the mere prevention of opportunistic behavior toward the dynamic alignment of clinical, organizational, and system-level objectives, acknowledging that health care value is co-produced by multiple actors across the patient care continuum. From this perspective, value-based contracts are best understood as inherently incomplete arrangements, whose effectiveness relies not only on financial incentives but also on governance structures, trust, organizational learning, and robust information infrastructures (8, 12).

Nevertheless, the literature indicates that the operationalization of VBHC has, in practice, relied heavily on instruments derived from incentive economics, including performance-based payment models, standardized quality metrics, and bonus schemes tied to selected indicators. While such instruments are consistent with accountability and incentive-alignment logics, several authors argue that this operational translation may strain the original conceptual ambition of VBHC by shifting attention away from value generation across the full care cycle toward the attainment of short-term, measurable targets (8, 12, 32).

A critical limitation that cuts across the theoretical frameworks reviewed concerns the definition of value itself. Although the term is used extensively in both the academic and policy literature, it is rarely interrogated as a contested concept (6). The dominant operationalization, value as quality per unit cost, reflects a payer-centric vantage point grounded in efficiency and resource allocation logics (10). This formulation, most prominently associated with Porter and Teisberg (8, 32), is analytically tractable and well suited to the contractual and incentive-alignment frameworks that structure most reviewed studies. However, it does not exhaust what different stakeholders regard as valuable (6). Patients may prioritize outcomes that are difficult to quantify, such as dignity, continuity of care, or alignment with personal goals; clinicians may emphasize professional autonomy, clinical appropriateness, and relational dimensions of care (33); and provider organizations may weigh sustainability, workforce well-being, and equity considerations that fall outside payer-defined metrics (23, 34). Because most empirical studies in this review operationalize value through payer-aligned indicators, claims data, cost measures, or administratively defined quality metrics the evidence base is systematically skewed toward one stakeholder perspective (13). This has implications for how findings should be interpreted: improvements in measured value may not translate into meaningful gains for patients or frontline clinicians, and conversely, dimensions of value that matter to these actors may remain invisible within prevailing analytical frameworks (6, 35). Future research would benefit from explicitly adopting multi-stakeholder definitions of value and from developing outcome measures that reflect patient- and clinician-relevant priorities alongside payer efficiency concerns (16).

However, the limitations of principal–agent models extend beyond contract incompleteness and multitasking. A subset of studies included in this review incorporated insights from behavioral economics and cognitive psychology, challenging the assumption that providers and patients respond to incentives solely through rational economic calculations. These perspectives emphasize that decision-making is shaped by cognitive heuristics, framing effects, social norms, and bounded rationality. Concepts such as loss aversion, as described by Prospect Theory, suggest that individuals may respond differently to equivalent incentives depending on whether they are framed as gains or losses (36). Behavioral responses are further shaped by peer comparison, salience, and perceived fairness — all of which reflect the operation of cognitive heuristics: the mental shortcuts through which individuals evaluate complex situations under uncertainty (29).

The concept of heuristics is particularly relevant to understanding why value is appraised inconsistently across stakeholders. Physicians, patients, payers, and administrators each assess the value of a clinical intervention through a lens shaped by their individual experiences, professional identities, institutional roles, and prior beliefs. This subjectivity is not merely anecdotal: it reflects a structural feature of healthcare decision-making in which the same payment reform may be simultaneously perceived as a legitimate accountability mechanism by a payer and as an intrusive control device by a frontline clinician (6). Far from being noise around a rational signal, these divergent appraisals are systematic, shaped by the heuristics each actor uses to reduce the cognitive complexity of value assessment.

Incorporating this perspective into the analysis of payment model effects complements the insights from Prospect Theory and principal–agent models and helps explain why financial incentives alone are unlikely to produce uniform behavioral responses across diverse actors and settings. These mechanisms, heuristic reasoning, framing effects, and bounded rationality connect directly to the motivational dynamics explored in Domain 2.

Similarly, the multitasking problem highlights that actors may concentrate effort on incentivized activities at the expense of non-measured dimensions of care, whereas motivational theories suggest that externally imposed incentives can interact with professional values and intrinsic motivations in complex ways (29, 37). Together, these perspectives help explain why payment reforms that rely on similar financial mechanisms frequently produce heterogeneous results across settings and why improvements in incentivized indicators do not necessarily translate into broader value creation.

### 4.2 Domain 2—core theoretical mechanisms: how incentives produce effects

Performance-based and value-based payment models rest on the assumption that financial incentives shape provider behavior by linking remuneration to specific actions or outcomes. Empirical evidence shows that these incentives are associated with measurable changes in professional practice, particularly in process measures, adherence to clinical guidelines, and documentation. Across the included studies, positive effects are more consistent for process indicators and easily measurable activities: P4P programs have demonstrated statistically significant improvements in incentivized prescribing and clinical interventions (20), while systematic reviews report modest but recurrent gains in protocol adherence and process indicators, with marked variation across settings (19, 21).

The evidence also documents negative results and adverse consequences, particularly when program design intensifies strategic behavior. Systematic reviews and empirical analyses report substantial heterogeneity of effects, limited or no impact on final clinical outcomes, the occurrence of gaming, and the displacement of priorities toward incentivized activities (21, 22), as well as rapid organizational responses focused primarily on what is measured (38).

There is also evidence suggesting adverse distributive effects, including limited reductions in health inequalities and poorer relative performance among providers serving more vulnerable populations, indicating risks to equity when incentive schemes fail to incorporate adequate risk adjustment (39, 40). In specific contexts, the introduction of P4P has been associated with mixed effects, combining increases in desired service utilization (e.g., institutional deliveries) with perceived adverse consequences for care provision and the work experience of health professionals (41), including perceptions of distributive and procedural injustice in bonus allocation, with the potential to undermine engagement (42).

From the perspective of principal–agent theory, the positive effects observed in part of the literature arise from a reduction in the misalignment between principals' objectives and agents' utility functions, enabled by greater observability and contractualization of performance. Empirical studies indicate that linking remuneration to indicators of quality, efficiency, or clinical outcomes helps redirect provider effort toward behaviors that were previously underemphasized under volume-based payment models, promoting greater adherence to protocols, standardization of practices, and a focus on monitorable results (19, 20). However, the literature consistently shows that the magnitude and direction of behavioral responses vary substantially across contexts, reflecting differences in incentive design, baseline provider performance, the financial intensity of programs, and organizational capacities for monitoring and implementation (12, 21, 43). In addition, authors report unintended negative effects, including strategic behavior, reallocation of effort toward incentivized indicators, neglect of non-measured dimensions of care, and potential adverse impacts on equity, particularly in settings serving more vulnerable populations (19, 23).

From the Value-Based Health Care (VBHC) perspective, these effects are interpreted as evidence that changing the payment model can contribute to reorienting the system toward value creation by aligning economic incentives with patient-relevant outcomes. Authors who explicitly adopt the VBHC framework argue that performance-based models promote greater organizational focus on outcomes, transparency, and accountability, particularly when combined with consistent measurement and monitoring strategies (16, 21, 32). However, these authors also acknowledge that observed gains tend to concentrate on dimensions that are readily measurable in the short term (19–21, 43). Within the VBHC perspective, bundled payments are the most frequently considered remuneration model. In this context, the reviewed literature identifies a consistent set of practical implementation challenges that limit their expected effects on behavior and performance. Although conceptually designed to align incentives across the full cycle of care and to stimulate care coordination, empirical studies indicate that their operationalization faces substantial challenges related to defining the episode of care, allocating responsibilities among multiple providers, and adequately measuring costs and outcomes (44).

From a principal–agent theory perspective, these challenges increase contractual incompleteness and heighten the risk of strategic behaviors, such as patient selection (cream-skimming), cost shifting outside the contracted episode, and excessive focus on more controllable stages of care (20, 21). In addition, the literature indicates that the behavioral effects of bundled payments are highly contingent on organizational capacity for clinical coordination, information integration, and financial risk management. In settings where such capacities are limited, bundles tend to function more as cost-containment instruments than as effective mechanisms for value creation, eliciting defensive responses from providers. From a motivational perspective, some studies suggest that the financial risk transfer associated with bundles can create incentive ambiguity at the professional level, particularly when physicians and care teams perceive limited control over the factors determining episode costs and outcomes. Under these conditions, economic incentives may weaken intrinsic motivation and induce risk-avoidant behaviors, to the detriment of clinical innovation and personalized care (21).

Another central mechanism discussed in the literature concerns the interaction between extrinsic incentives and intrinsic motivation. Studies grounded in behavioral economics and motivation theories document that motivation crowding-out, introduced in Domain 1, manifests empirically as compliance-oriented behavior, reduced professional engagement, and gaming practices, to the detriment of substantive improvements in care quality (20, 22, 37). The risk is particularly high when incentives are perceived as controlling, poorly aligned with professional values, or disconnected from outcomes that are clinically meaningful to patients (28, 41, 42).

The literature also distinguishes alternative reward logics within performance-based payment models, particularly the distinction between incentives based on achievement (absolute performance level) and improvement (relative improvement compared to prior performance). Empirical evidence indicates that this design choice is central to the behavioral responses observed (12, 19). Programs such as the Hospital Value-Based Purchasing initiative of the Centers for Medicare & Medicaid Services (CMS) explicitly combine both approaches, rewarding absolute performance and performance improvement over time to balance incentives for excellence with incentives for incremental improvement (20, 43). From a theoretical perspective, achievement-based incentives tend to favor organizations with greater installed capacity, stronger infrastructure, and a history of high performance, whereas improvement-oriented incentives are more effective in engaging providers with lower baseline performance by reducing barriers to participation and increasing the perceived attainability of targets (12, 21). In this regard, the literature highlights that an improvement-based logic may mitigate regressive effects and promote greater equity, particularly in settings where providers serve more vulnerable populations; by contrast, an exclusive emphasis on achievement may exacerbate inequalities by concentrating resources among already well-positioned organizations (19, 43).

### 4.3 Domain 3—from incentives to care redesign: theoretical integrations in value-based health care

The reviewed literature conceptualizes pay-for-performance (P4P) as a contractual instrument designed to induce provider behavior, whose effectiveness depends on its embedding within organizational arrangements capable of sustaining changes in clinical practices and care processes (28, 45–47). Qualitative evidence indicates that P4P reshapes professional routines and boundaries by shifting clinical work toward measurable targets, thereby generating organizational effects that extend beyond the individual provider level (38, 48). Empirical studies show that the translation of P4P into care improvement is mediated by organizational capacities for information management and data-driven decision-making, without which incentives tend to operate in fragmented ways and yield limited effects on value creation (28, 49). The integration of P4P with chronic care–oriented models and disease management strategies is identified as a necessary condition for financial incentives to produce sustained effects over time, as such models reinforce multidisciplinary coordination, longitudinal follow-up, and standardization of clinical practices (45, 47).

Within the Value-Based Health Care (VBHC) framework, studies characterize VBHC as an organizational paradigm that requires the reorganization of care along the patient journey and the systematic measurement of outcomes and costs, thereby aligning VBHC with care models oriented toward continuity and coordination (50, 51). Empirical evidence suggests that VBHC implementation is associated with organizational learning processes and changes in clinical routines, in which observed effects derive from the articulation between new payment arrangements and care redesign strategies grounded in the Chronic Care Model and principles of continuity of care (14, 33, 52). The literature further emphasizes that operationalizing VBHC depends on integrating cost and outcome measurement along the patient pathway, which has been supported by activity- and time-based costing approaches, particularly Time-Driven Activity-Based Costing (TDABC), as a foundation for value-oriented clinical and managerial decision-making (53, 54). Recent implementation research further suggests that these measurement capabilities are necessary but not sufficient for successful payment reform. Evidence from alternative payment models indicates that implementation also depends on physician leadership, benchmarking systems, robust data infrastructures, stakeholder engagement, and regulatory environments capable of supporting organizational transformation, reinforcing that value-based payment arrangements should be understood as organizational innovations rather than merely financial contracts (55).

The reviewed studies also link value-based models to Population Health Management strategies and the Quadruple Aim framework, an expansion of the original Triple Aim that incorporates the well-being of the health workforce as a fourth performance dimension (56) by extending the notion of value beyond single episodes of care to encompass population-level outcomes, patient experience, financial sustainability, and clinician well-being as integrated performance dimensions (24, 57). Evidence indicates that contexts in which P4P and VBHC instruments are combined with population health management strategies and longitudinal care coordination tend to exhibit more consistent effects that are less confined to isolated process indicators, although such effects remain contingent on organizational capacities for data integration, clinical governance, workforce engagement, and financial risk management (25, 35, 58).

### 4.4 Domain 4—levels of analysis and institutional context

The reviewed literature indicates that the effects of pay-for-performance and value-based payment models vary systematically across levels of analysis and according to the institutional contexts in which incentives are implemented. At the professional level, studies show that physicians and care teams respond heterogeneously to incentives, with behavioral responses mediated by professional norms, perceptions of autonomy, ethical values, and occupational identities, which condition both uptake and resistance to performance-based payment arrangements (38, 51, 59). These responses are not reducible to rational economic calculation: cognitive heuristics the mental shortcuts through which individuals evaluate complex situations under uncertainty shape how providers interpret, frame, and respond to incentive structures. Physicians may apply availability or representativeness heuristics when assessing the relevance of a performance indicator to their clinical context and may respond differently to equivalent incentive designs depending on how targets are framed, who communicates them, and what peer comparison signals are embedded in the program. These heuristic-driven responses help explain why behavioral effects remain heterogeneous even when the financial structure of incentives is held constant across settings (60, 61).

At the organizational level, the capacity to respond to incentives depends on governance structures, leadership, information systems, and organizational maturity in performance monitoring and care coordination. Qualitative evidence suggests that implementing value-based models requires institutional work to accommodate new logics of accountability and measurement within professional organizations characterized by hybrid governance arrangements that combine managerial and clinical logics (21, 28, 58). Studies also indicate that accountability instruments inspired by New Public Management reconfigure organizational routines and internal power relations, with ambiguous effects on professional autonomy and clinical–organizational alignment (38, 62).

At the health system level, the effects of incentive-based payment models are conditioned by broader institutional arrangements, including financing regimes, regulatory structures, service market architectures, and policy legacies. Comparative evidence suggests that pay-for-performance and value-based payment reforms follow distinct trajectories of diffusion and institutionalization across health systems, reflecting differences in state capacity, interorganizational coordination, and regulatory coherence (47).

At the level of payers and regulators, studies highlight that incentive design is strongly shaped by policy choices, governance structures, and institutional capacities for monitoring and enforcement. Evidence suggests that limitations in strategic purchasing, power asymmetries between payers and providers, and regulatory coordination failures constrain the transformative potential of value-based payment models, frequently resulting in hybrid arrangements that combine cost-containment logics with partial quality incentives (24, 25). The literature also emphasizes that equity considerations, social risk adjustment, and distributive justice concerns influence the design and effects of incentives at the regulatory level, conditioning their impact on disparities in access and health outcomes (34, 39, 40).

Across levels, the reviewed studies converge in showing that pay-for-performance and value-based payment models function as institutionally mediated governance instruments whose effects depend on interactions between levels of analysis and institutional contexts, including hybrid organizational governance, legacies of New Public Management, and the political economy of health systems. Consequently, the effects of incentives are not intrinsic to contractual instruments but emerge from how they are interpreted, implemented, and negotiated within specific organizational and institutional environments (58, 63).

### 4.5 Domain 5—limits, critiques, and normative tensions

The reviewed literature problematizes pay-for-performance and value-based payment models through multiple normative and critical lenses, indicating that their adoption cannot be understood in purely instrumental terms. From the perspective of virtue ethics, studies argue that reducing clinical performance to contractual metrics may undermine core professional values such as practical wisdom, moral commitment to patients, and context-sensitive clinical judgment by shifting motivation from care-oriented goals toward target compliance (64, 65). Empirical evidence suggests that financial incentives can reshape the moral meaning of clinical practice, with potential risks to the intrinsic ethical responsibilities of health professionals (38, 64).

Principles of bioethics provide an additional lens by highlighting potential tensions between performance-based payment models and the principles of autonomy, beneficence, nonmaleficence, and justice, particularly when financial incentives influence sensitive clinical decisions or prioritize organizational targets over patients' preferences and needs (65, 66). The literature indicates that, in contexts where incentives shape decisions related to screening, treatment choices, or episode definitions, there are risks of compromising patient autonomy and creating conflicts of interest in clinical practice (65).

From the standpoint of distributive justice, studies indicate that pay-for-performance and value-based payment models may reproduce or exacerbate inequalities, particularly when social risk adjustment and structural constraints faced by providers are insufficiently incorporated into incentive design. Empirical evidence suggests that P4P programs may be less effective in settings serving socially vulnerable populations, with the potential to penalize organizations operating in contexts of higher socioeconomic deprivation (34, 39, 40). The literature further notes that performance-based resource allocation may generate regressive effects by concentrating financial rewards among organizations with greater baseline capacity and structural advantages (34, 39).

From a political economy of health perspective and critiques of healthcare commodification, studies problematize the incorporation of market-oriented logics and performance management into health systems, highlighting risks of reframing care as a commodity and subordinating public and professional values to efficiency metrics and economic returns (24, 25). Evidence indicates that failures in strategic purchasing and governance arrangements may reduce value-based payment models to cost-containment instruments, thereby undermining their normative promise of promoting patient-centered care (54).

Critiques grounded in the performativity of metrics emphasize that indicators do not merely measure reality but actively shape it. Beyond the behavioral distortions documented in Domain 2, this performative logic carries normative consequences: by rendering certain dimensions of care measurable and others invisible, metric systems implicitly assign moral weight to efficiency over relational care, interprofessional coordination, and responsiveness to complex patient needs (28, 38, 48).

Taken together, these normative and theoretical critiques demonstrate that VBHC and P4P are not neutral policy instruments but institutional arrangements that reshape professional values, power relations, clinical practices, and distributive dynamics. Incorporating these critical lenses is essential to avoid naïve instrumental readings of incentive-based reforms and to inform policy designs that reconcile efficiency and value creation with ethical and distributive commitments.

## 5 Conclusion

This review demonstrates that the gap between P4P and VBHC is not merely conceptual but institutional and political: while both share a commitment to aligning provider incentives with value creation, their effective articulation remains contingent on conditions that are rarely met simultaneously in practice — adequate governance capacity, longitudinal care coordination infrastructure, multi-stakeholder definitions of value, and explicit equity safeguards. The five analytical domains examined here reveal a persistent tension between the contractual logic of performance-based payment, which operates through measurable and short-term targets, and the organizational and normative ambition of VBHC, which requires care redesign, workforce engagement, and the integration of costs and outcomes across the full patient journey. Bridging this gap demands more than financial engineering: it requires treating payment reform as an institutional project embedded in the political economy of each health system.

This review makes an original contribution by providing, for the first time in the literature, a systematic mapping of the full landscape of theoretical lenses applied to value-based and performance-based payment models across diverse health systems — integrating economic, organizational, institutional, behavioral, and normative perspectives that have previously been developed in separate disciplinary traditions. Unlike impact evaluations or program assessments, this synthesis enables cross-theoretical comparison of the assumptions, causal mechanisms, and contextual conditions underlying incentive-based payment reform, offering a conceptual foundation for future research design and policy analysis.

Taken together, the five analytical domains converge on a central finding: the behavioral distortions documented in Domain 2 gaming, priority shifting, crowding-out and the normative tensions identified in Domain 5 equity trade-offs, commodification risks, threats to professional autonomy are not design failures of specific programs but structural features of incentive-based governance when decoupled from adequate institutional and ethical foundations. The well-documented gap between improvements in process indicators and demonstrable gains in final clinical outcomes reflects this deeper problem: when payment models operate through contractual and metric logics alone, without integration into care delivery models, governance arrangements, and multi-stakeholder definitions of value, they tend to produce measurement gains rather than meaningful value creation.

From a normative perspective, the incorporation of critical lenses virtue ethics, principles bioethics, theories of distributive justice, critiques of healthcare commodification, and metric performativity demonstrates that incentive-based reforms reconfigure professional values, power relations, and clinical practices, producing tensions between efficiency, accountability, and ethical and distributive commitments. These findings underscore the need to move beyond naïve instrumental readings of VBHC and P4P and to recognize them as complex institutional arrangements whose effects depend on their integration with care models, organizational governance, and normative frameworks oriented toward equity and patient-centered care.

This review has important limitations. First, although the analyzed corpus is broad, heterogeneity in study designs and institutional contexts limits direct comparability of findings and the possibility of strong causal inference. Second, the predominance of observational and qualitative studies in some domains may introduce selection and publication biases and constrain generalizability. Third, the operationalization of “value in health care” varies substantially across studies, hindering quantitative synthesis of effects and reinforcing the interpretive nature of the present analysis. Fourth, the reviewed literature is heavily concentrated in North American and Western European health systems, which limits the transferability of findings to low- and middle-income settings and to health systems with distinct financing structures, regulatory traditions, and workforce conditions. Finally, the review depends on the availability and quality of reported data in the included studies and may underrepresent implementation experiences in low- and middle-income settings or initiatives not published in indexed journals. No formal critical appraisal of methodological quality was conducted, as the objective was interpretive and theory-oriented rather than effect estimation.

The evidence reviewed suggests three interconnected priorities for researchers and policymakers. First, future studies should prioritize the measurement of final clinical outcomes, mortality, disease burden, functional status, and patient-reported outcomes, rather than relying predominantly on process indicators, to assess whether incentive-based reforms produce clinically meaningful gains rather than measurement artifacts. Second, research should expand beyond predominantly North American and Northern European contexts: of the 76 studies included in this review, fewer than 8% were conducted in low- and middle-income countries, with Brazil, Tanzania, and China accounting for the entirety of this representation. The governance conditions, financing arrangements, and professional cultures that modulate the effects of P4P and VBHC differ substantially across health systems, and evidence from Latin American, sub-Saharan African, and Southeast Asian settings, where universal coverage reforms are actively underway, remains critically scarce and is needed to inform context-sensitive policy design. Third, policymakers should treat payment reforms as institutional and ethical projects rather than financial engineering tools. Beyond the design of financial incentives, successful implementation requires organizational capabilities that support practice transformation, including clinical governance, physician leadership, interoperable data infrastructures, benchmarking systems, stakeholder engagement, and longitudinal care coordination. Recent implementation research on alternative payment models reinforces that these organizational capabilities are essential for translating value-based payment principles into sustainable practice change across different health system contexts (55). The reviewed evidence consistently shows that coherent effects emerge only where these implementation capabilities are coupled with explicit equity safeguards and attention to workforce well-being as a performance dimension, consistent with the Quadruple Aim.

## Data Availability

The original contributions presented in the study are included in the article/Supplementary Material, further inquiries can be directed to the corresponding author.
